# ‘I am not a depressed person’: How identity conflict affects help-seeking rates for major depressive disorder

**DOI:** 10.1186/1471-244X-12-164

**Published:** 2012-10-02

**Authors:** Caroline Farmer, Paul Farrand, Heather O’Mahen

**Affiliations:** 1 Mood Disorders Centre, University of Exeter, Exeter, UK

**Keywords:** Help-seeking, Depression, Goals, Goal conflict, Identity, Identity conflict, Barriers to treatment

## Abstract

**Background:**

There is a significant treatment gap for patients with depression. A third of sufferers never seek help, and the vast majority of those who do only do so after considerable delay. Little is understood regarding poor help-seeking rates amongst people with depression, with existing research mainly focussed on the impact of barriers to treatment. The current study explored psychological factors affecting help-seeking behaviour in clinically depressed individuals.

**Methods:**

Semi-structured interviews were conducted with 20 current or previously clinically depressed participants who either had or had not sought professional help. Thematic analysis was used to analyse results.

**Results:**

The onset of depressive symptoms created conflict with participants’ identity and personal goals. Delays in seeking help were primarily attributed to the desire to protect identity and goals from the threat of depressive symptoms. Participants used avoidance strategies to reduce the perceived threat of depressive symptoms on identity. These strategies interfered with help-seeking. Help-seeking was only undertaken once participants reached a point of acceptance and began to make concessions in their identity and goals, at which time they reduced their use of avoidance.

**Conclusions:**

Difficulties resolving conflict between identity and depressive symptoms may account for significant delays in seeking help for depression. The results have implications for predicting health behaviour and improving treatment uptake for depression, and may inform existing help-seeking models.

## Background

Major depressive disorder (MDD) affects between 10-15% of people over the course of their lives, and is expected to become the second leading cause of disability in the world by 2020 [1,2]. Depressive symptoms lead to ill health, increased mortality and have a significant impact upon social and occupational functioning [3]. Improved understanding and treatment of MDD has therefore become a significant research priority [4].

Despite the strong evidence base for the effective treatment of depression with antidepressant medication and psychological therapies e.g. CBT, IPT [5-7], efforts to treat depression are undermined by poor help-seeking rates. Between a third and a half of those with depression do not seek treatment [8]. Furthermore, less than half of all patients seek help within the first year of symptoms [9], despite quality of life reductions occurring immediately in the transition between normal and low mood [10]. Average help-seeking delays for depression are as high as eight years [8,11,12]. There is growing evidence that these long delays in seeking help contribute significantly to the unmet need for treatment, and contribute to increased illness length and higher symptom severity at the start of treatment [13].

The reasons underlying such long delays in seeking treatment for depression are not well understood. The majority of the research to date has highlighted barriers to treatment that may influence cost-benefit decisions about seeking help. These include attitudinal (beliefs and attitudes towards mental illness and treatment), structural (lack of time, cost and treatment availability), and knowledge barriers (patient knowledge of illness and treatment availability) [14]. Overall, research suggests that attitudes towards mental health and treatment are the most influential of these in predicting help-seeking intentions and behaviour [15-21]. As a consequence, models driven by the assumption that health care decisions are driven by a rational analysis of costs and benefits have been criticised for not putting enough emphasis on the psychological and emotional influences involved in seeking help [22].

Despite the evidence supporting the role of psychological factors in influencing help-seeking, there has been surprisingly little research exploring how these factors influence help-seeking for depression [23]. A significant proportion of people with depression who have not sought help report they perceive no need for treatment [18,20,24]. Previous studies also suggest that up to two thirds of the delay in seeking help for depression may be due to individuals not recognising the cause of their symptoms [13,14,25]. Furthermore, individuals often express a preference to manage their symptoms alone [11,14,17,19], and suggest that seeking help for their symptoms would negatively impact their self-esteem [26]. Research is therefore needed to explain how individuals recognise and explain changes in their mood that occur with the onset of depression, and how the interpretation of mood influences decisions to seek help. To further our understanding of help-seeking decisions, research which explores the patients’ perspective is essential [27]. This study employed a qualitative methodology to provide a detailed account of individuals’ experiences following the onset of depressive symptoms. Interviews were conducted with currently and formally depressed individuals. The analysis sought to identify common factors that influenced participants’ decisions to seek treatment for depression or that contributed to help-seeking delays. Further understanding of these issues can be used to identify important influences on help-seeking rates, and improve appropriate and earlier help-seeking in depressed individuals.

## Methods

### Recruitment

Participants were included in the study if they either had current Major Depressive Disorder (MDD), or met diagnostic criteria for MDD within the 12-months prior to interview. Recruitment procedures sought to include both people who had, and people who had not, sought help for depression. Various recruitment methods were used; an article placed in a local newspaper, advertising the study via a local self-help support group for depression, a general university email distribution list for people interested in research participation, and via a social networking website. As previous research has indicated that not all people with depression may recognise the cause of their symptoms [13,14,25], adverts specified an interest in also talking to people who had experienced a period of pervasive low mood. It was also stressed that a diagnosis of depression or the receipt of treatment was not required.

### Procedure

Interested participants followed a web link in adverts that directed them to further information about the study. Those consenting to participate were then directed to an online questionnaire that included the PHQ9 [28] to screen for current depressive symptoms and questions about past episodes of low mood and past help-seeking for low mood.

Respondents who indicated that they had experienced a period of pervasive low mood within the previous 12-months, or scored 10 and above on the PHQ9 (indicating current symptoms of depression), were contacted to participate in the interview.

Interviews were conducted over the telephone or in person, based on participant preference. To determine the presence or absence of current and/or past episodes of major depression, participants were administered the screening component and the mood section of the SCID I [29] at the start of the interview. The SCID I is a structured interview schedule that has been found to have good reliability and validity in the diagnosis of Axis I disorders. Participants who gave positive responses to items in the screening component were asked to complete the mood disorders section of the SCID I. Participants who completed the mood disorders module of the SCID I were discussed within the research team to establish whether depression was the primary diagnosis. Author HO is a clinical psychologist, PF is an accredited CBT therapist. All participants who met SCID-I criteria for current MDD or MDD in the past 12-months completed the semi-structured qualitative interview.

### Sample

**Figure 1:**
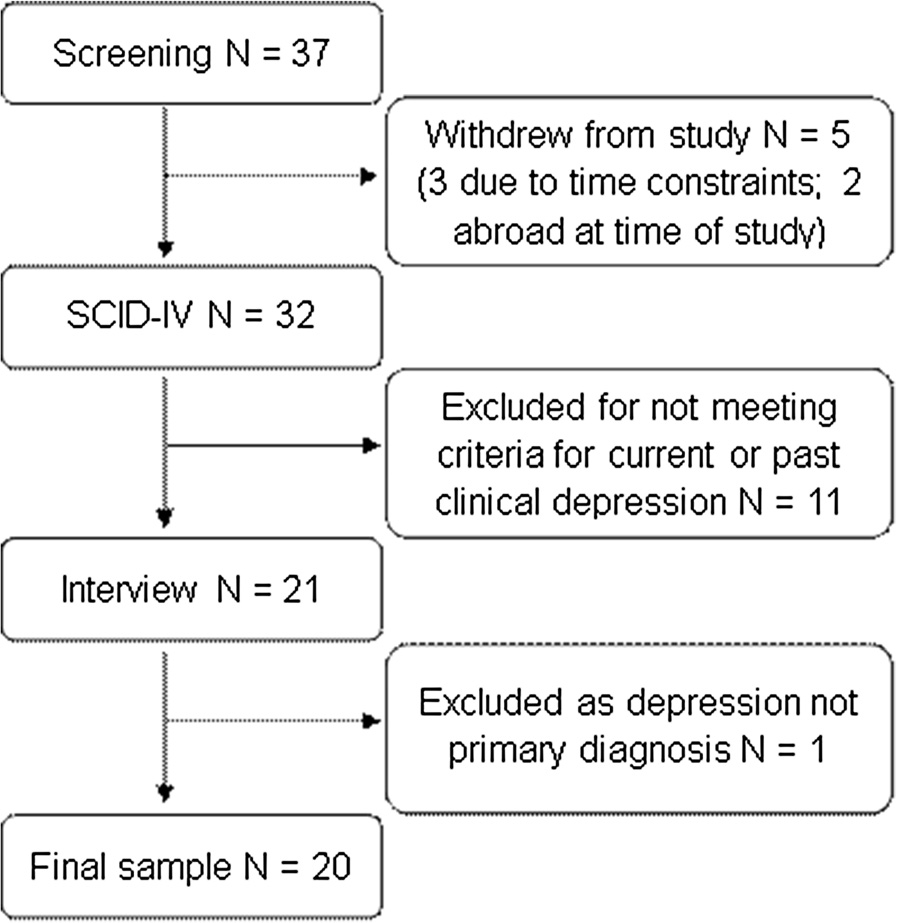
Participant recruitment.

Figure 1 displays the progress of participants through the recruitment for the study. A final sample of twenty participants (17 female, 3 male) aged between 18–69 years (mean = 41.8, SD = 19.2) were included in the analysis.

### Interview schedule

A semi-structured interview schedule was developed by the research team with the goal of understanding participants’ help-seeking decisions following symptom onset. The schedule was based on a review of the literature and the clinical experience of the research team, and was guided by the following questions; ‘can you tell me about the time in which you started to notice a change in your mood?’, ‘how have/had you been coping with symptoms over this period of time?’ and ‘can you explain the decision-making process you went through in choosing to seek/not seek help?’. The interview schedule was modified iteratively, as the interviews and concurrent data analysis proceeded, to incorporate new information and focus progressively on emerging themes. As participants described some difficulty in recognising and labelling their symptoms as depression, additional questions about how participants recognised and interpreted their symptoms were included in the interview schedule. As avoidance of symptoms began to emerge as a strong theme, the schedule was further modified to incorporate further questions about coping strategies participants were using prior to seeking help. Further probing of factors that influenced participants’ decisions to seek help were also included to give more depth to the transcripts.

Interviews were conducted by CF following guidance by Weiss [30], and lasted between 40–75 minutes. All interviews were recorded and transcribed verbatim. A portion of each transcript (approximately 10%) was reviewed for accuracy following transcription.

### Data analysis

We employed a thematic analysis approach [31], drawing on principles of grounded theory to identify themes within participants’ accounts of their decisions to seek help for depression. This included constant comparison of participant transcripts, concurrent with data collection and identification of themes from the data. Study findings were developed through a group consensus process within the research team (2 psychologists, 1 clinical doctoral student). This process enhanced data interpretation. We began by developing codes from the raw data, based on common themes identified as analytically relevant to addressing the study research questions. As coding development proceeded, code definitions were influenced by useful concepts from the literature, in addition to those emerging from the data. Interview transcripts were read by CF. A coding reliability check was provided by HO and PF, who independently read and coded transcripts. An iterative process was used to compare results until agreement was reached on code definitions and application of codes to interviews. The final coded transcripts were entered into NVIVO8 software [32] to assist with data analysis, with code reports produced and summarised by the investigators. The research team met regularly to review the code summaries and discuss and interpret the data in light of the original study purpose, with a focus on informing the process of help-seeking in persons who have experienced clinical depression.

### Ethical considerations

This study was approved by the University of Exeter ethics committee, and complied with British Psychological Society Code of Conduct and the University of Exeter procedures for data protection. Informed consent for participation in the study was obtained from each participant. All depressed participants, including three participants who had not sought help at the time of the interview, were offered information on formal and informal sources of support for MDD following completion of the study.

## Results

### Demographics

Twelve participants met criteria for MDD and eight participants had MDD within the past 12-months. Three participants had not ever sought help. Thirteen participants had experienced more than one episode of depression. Eleven of these participants failed to seek help for at least one of their previous episodes of MDD. Fourteen participants reported experiencing symptoms of a comorbid psychological disorder, most often anxiety-related, occurring at the time that they were depressed.

Help-seeking delays ranged between 2 weeks and 30 years for participants’ most recent episode of depression (Mean = 8.24 years; SD = 9.10). Four participants reported help-seeking delays of longer than 10 years. Participants (N = 7) who had sought help for at least one previous episode of depression sought help more quickly in their most recent depressive episode (Mean delay = 4.28 years, SD = 7.27) than those who had no history of help-seeking (Mean delay = 8.06 years, SD = 8.78). Of all participants, only one participant who met diagnostic criteria for depression had not recognised that her symptoms were caused by depression at the time of interview.

### Qualitative analysis

Three themes emerged regarding participants’ accounts of their help-seeking decisions (see Table 1). These themes were; ‘Depressive symptoms create conflict’, ‘Avoidance’, and ‘Acceptance’. Two disconfirming cases in ‘Avoidance’ are also discussed.

**Table 1:** Description of common themes

**Themes**	**Description**
DEPRESSIVE SYMPTOMS CREATE CONFLICT	Symptoms are identified as abnormal. Participants express fears that symptoms threaten their identity and important goals
AVOIDANCE	Participants cope with identity and goal conflict by avoiding their symptoms and using psychological strategies to reduce the perceived threat to goals. The avoidance of symptoms was the primary cause of help-seeking delay
ACCEPTANCE	Participants stop avoiding their symptoms and begin to make decisions about seeking help. Identity and goals begin to change to accommodate the experience of depression.

**Figure 2:**
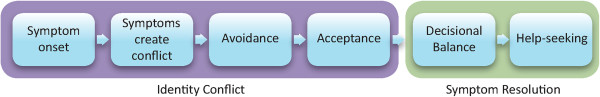
The progression of themes over time.

As illustrated in Figure 2, the three themes were found to appear sequentially across participants’ accounts. Following symptom onset, participants described feeling that their DEPRESSIVE SYMPTOMS CREATED CONFLICT with their identity and the goals they wanted to achieve. The onset of conflict prompted the use of AVOIDANCE strategies, which extended help-seeking delays. ACCEPTANCE represented the end of avoidance strategies, and the beginning of cost-benefit decisions to seek help for depression.

#### Theme 1 – Depressive symptoms create conflict

Participants discussed becoming aware of symptoms approximately two weeks after onset. This included recognition that their mood, thoughts and behaviour had changed and were impacting on their functioning, however they did not necessarily label their symptoms as depression at that time. Participants recognised the onset of their symptoms by comparing their current state with their past self, and by making comparisons with those around them. These comparisons were important in symptom recognition, as they helped participants to decide whether the change they were experiencing was ‘normal’.

“I felt really awful and I thought this can’t be how people feel normally when they are a bit sad” (Female 2)

Recognising that symptoms were abnormal was an essential stage prior to seeking help. There were a small number of participants who failed to notice their symptoms were abnormal, and therefore experienced extended help-seeking delays.

“I didn’t really talk to people…so to me that was normal and I didn’t I suppose realise that feeling unhappy wasn’t necessarily a normal way of life” (Female 15)

The recognition that participants were experiencing an abnormal change in functioning was highly distressing. This distress was primarily caused by concerns that symptoms threatened participants’ identity and their related goals. In particular, participants worried about the impact of this change on their identity, reporting concerns that they were becoming someone they did not want to be.

“I want to be a doctor and I want to be seen as someone who could do it as opposed to people look at me and go ‘oh no, she has problems’” (Female 8)

“I’m not a control freak but I do like to know that I’m in charge…But of course when you’re depressed you’re not, and facing up to that was something I didn’t like having to do”(Female 7)

The type of symptoms experienced early in their depression influenced the speed with which participants recognised their symptoms as abnormal. Participants found it easier to compare changes to their behaviour as a result of physical symptoms with the observable behaviour of others. However, participants felt much less knowledgeable about whether changes to their thoughts and emotions were normal, finding these much more difficult to compare. As a consequence, those participants who reported fewer physical symptoms at the beginning of the illness experienced longer delays in symptom recognition.

#### Theme 2 – Avoidance

The onset of depressive symptoms was interpreted by participants as a threat to their identity. Acknowledging symptoms, and acting to resolve them, involved accepting that their identity had changed in a negative way. Rather than accept this change, participants discussed how avoiding symptoms allowed them to reduce the perceived threat of symptoms.

“*I think to me depression was kind of, being weak, wallowing and just being generally mental, crazy, and I just, I’m not any of those, I’m perfectly fine*” *(Female 6)*

Participants used distraction to ignore their symptoms and their impact on functioning. In addition, participants used psychological mechanisms to reduce the perceived threat of symptoms. This included misattribution (blaming symptoms on external, short-term causes), and bargaining (ignoring deficits in functioning in favour of evidence that symptoms were normal).

*“you think well, something’s not right here…and then you kind of turn and think ‘oh it must be the course’, ‘it must be the group of people’…not ‘why do I feel like this, let’s go find out’. It’s there’s something wrong with the situation as opposed to me”* [misattribution] *(Female 3)*

*“I kept saying ‘there is nothing wrong with me…look I’m working all these hours, what can be wrong with me’”* [bargaining] *(Female 15)*

Reducing conflict through avoidance led to a short-term reduction in emotional distress. However, participants reported that this was only temporary and did not resolve their symptoms. As symptoms increased in severity, participants found it more difficult to use distraction, and misattribution and bargaining strategies became more difficult to justify. Avoidance strategies would also frequently fail when environmental cues (e.g. comments by friends and family) would remind participants of their symptoms and their reduced functioning.

‘when I had depression…it was easier if people didn’t ask me: “How are you now, are you feeling better?”… I used to find that a very…unhelpful question’ (Male 1)

#### Disconfirming cases

Three participants were able to resolve identity conflict sooner than other participants in the sample, reporting less use of avoidance strategies and much shorter help-seeking delays. These participants discussed how reduced conflict between symptoms and their identity led to less emotional distress, and facilitated their decision to seek help sooner. Significant others were central in reducing conflict for all three participants, although in different ways.

Two of the participants discussed how they received non-judgemental support and reassurance from significant others after disclosing their symptoms. This support gave them confidence that their identity and related goals could be retained despite their symptoms, therefore reducing conflict.

For the third participant, it was the unsupportive response of significant others that facilitated a reduction in identity conflict. Following the onset of symptoms, this participant’s family were highly critical of the change in her mood and behaviour, blaming her for her symptoms, and accusing her of being a weak and lazy person. This participant discussed how being someone with depression was a more positive identity than being a weak and lazy person. In this case, symptoms of depression became congruent with pursuing a more positive identity, and therefore reduced the conflict between symptoms and goals.

#### Theme 3 – Acceptance

Avoidance became more difficult as symptoms worsened, leading to rising levels of emotional distress. Increasing symptoms also led to further reductions in functioning, which created more conflict between symptoms and participants’ identity and related goals. At a time when levels of conflict and emotional distress were at their highest, participants often discussed reaching a point of acceptance towards their symptoms. Participants acknowledged that they may no longer be able to achieve some of their goals, and often expressed feeling a subsequent loss or confusion about their identity. Acceptance represented a large reduction in avoidance coping, and it was often at this time that participants discussed an acknowledgement that they were depressed.

“it just got to the point I just thought Christ enough is enough, if I don’t do something about this or find out…why it’s happening then I’m gonna go round this circle forever in my life, I’m not gonna have any friends, I’m not gonna get anywhere in my life”(Female 3)

“it just wasn’t who I wanted to be so yeh that was the trigger, that was when I decided I’d go and sort it out”(Female 12)

Acceptance also emerged at times when a particularly important goal was threatened and the consequences of not seeking help increased suddenly. These events increased the incentive for participants to resolve their symptoms so as to best pursue their goals. Most participants reported a negative cueing event (e.g. a partner threatening to leave), although occasionally positive events (e.g. a new job offer) were reported.

Following acceptance, participants discussed beginning to assimilate their symptoms into their identity.

‘I am sort of… finding myself again, you know over the last year, trying to get back to who I think I am’ (Female 3)

Participants discussed a clearer understanding of whether their short term goals would need to be abandoned or delayed while continuing to experience symptoms. In addition, uncertainty about how symptoms may affect long term goals and their identity was also a concern. Over time, longer-term goals were adjusted to accommodate for their experience with depressive symptoms. This was a long term transition, in most cases continuing after participants sought help.

Following acceptance, participants began to consider actions that could resolve their symptoms. Most participants felt that seeking help from their own doctor was the most suitable course of action, and considered this before alternatives such as local support groups, self-help and self-education.

Decisions about help-seeking were guided by the weighing of costs and benefits of seeking help, including the consideration of various barriers to help and beliefs about depression. Common barriers reported by participants incorporated attitudinal, structural and knowledge barriers (displayed in Table 2). Barriers were often interrelated, for example beliefs about the efficacy of treatment were informed by participants’ knowledge of MDD and treatment. At times when the weighing of costs and benefits did not support help-seeking or other action, participants returned to using avoidance coping to manage their symptoms.

**Table 2:** Factors influencing help-seeking decisions at the decisional balance stage

		**N participants**
Knowledge and attitudes to treatment	Beliefs about the appropriateness of treatment	12 (60%)
	Beliefs about the availability and accessibility of treatment	9 (45%)
	Fears of an unhelpful or non-empathic response from doctor/medical professional	6 (30%)
	Beliefs about the effectiveness of treatment	5 (25%)
	Fears that seeking help/receiving treatment will be uncomfortable or distressing	4 (20%)
Depressive symptoms	Increased symptom severity influenced beliefs about the appropriateness of treatment, and increased the costs of not seeking help	10 (50%)
	General feelings of low self-worth and hopelessness	6 (30%)
Structural barriers	Time commitments	3 (15%)
	Finance	2 (10%)
Other	Expectations of social stigma	9 (45%)
	Stoic attitude-preference to manage symptoms alone	4 (20%)
	Other costs/benefits, e.g. fears about outcome for medical record, to achieve important goals	10 (50%)

Most participants who decided not to seek help at this point did eventually seek help when the costs and benefits of seeking help changed, for example when their symptoms worsened. However, two participants reported having made the decision to never seek help for their symptoms and expressed a lack of interest in receiving information about treatment options following the interview. This decision was based on having weighed the costs and benefits of seeking help, and concluding that their symptoms were permanent and treatment would be ineffective. Both participants discussed having adapted their identity and goal structures to account for ongoing depressive symptoms.

“I got use to the fact that it’s probably going to be a problem somewhere and over the years I have learnt to cope with it I suppose I…probably look at that now and see normal” (Male 2)

## Discussion

Existing theories of help-seeking for depression have had mixed results and emphasised logistical and practical rather than psychological factors affecting help-seeking [22,23,27]. This study took a patient-centred approach and sought to get direct accounts of the help-seeking process from individuals who had experienced depression. Our results suggest that help-seeking for depression is a two-stage process where the balance of costs and benefits for seeking help is preceded by an identity conflict process marked by the avoidance of depressive symptoms. Participant accounts indicated that attempts to manage conflict through avoidance accounted for a significant proportion of delays in help-seeking. We identified three themes in the analysis, which together describe the onset and resolution of conflict between participants’ sense of identity and their depressive symptoms prior to seeking help.

The results of this study support findings that the costs and benefits of seeking help influence individuals’ help-seeking decisions [14], and suggest that all three barriers (structural, knowledge and attitudinal) may be helpful targets for intervention. However, as participants in this study did not begin to consider the costs and benefits of seeking help until late in the help-seeking process these interventions may have limited impact on reducing help-seeking delays. Rather, they may be most applicable to a sub-section of depressed individuals who have reached acceptance about their symptoms, and therefore may already have some intention to act to resolve these symptoms.

In contrast to prior research that suggested that individuals may have difficulty in recognising the cause of their symptoms [13,14,25], this did not cause significant delays in this sample. When lack of symptom recognition did occur, it was most prevalent in those individuals who had fewer physical symptoms and therefore had greater difficulty comparing themselves against the functioning in others. Regardless, the majority of the delay before seeking help was due to the denial and avoidance of symptoms, used by participants to cope with identity conflict.

Our understanding of the conflict process described by participants may be informed by identity and goal theories. These theories propose that people are driven to maintain a positive and stable view of themselves, including a belief that they are self-reliant and capable [33-36]. They posit that individuals pursue goals that maintain this self-view, as well as working towards desired future identities [37,38]. Participants in this study discussed how experiencing depressive symptoms threatened their ability to perceive themselves in a positive way, both in terms of their short and long term goals, and the ways in which these related to their perceived identity. Participants described how they struggled with beliefs that they were incapable and weak, and worried that their symptoms would prevent them from achieving desired future goals. The threat symptoms posed to individuals’ identity was particularly distressing, and identity conflict was the primary cause of avoidance behaviour. Participants attempted to protect their identity by using strategies to deny or avoid their symptoms. This temporarily enabled them to preserve their identity, but resulted in help-seeking delays.

Participants used both behavioural (e.g. distraction) and cognitive techniques (e.g. misattribution and bargaining) to avoid their depressive symptoms during the delay period. These strategies have been commonly reported in experimental studies of individuals who experience a conflict between their beliefs and behaviour [35,39-41] and are consistent with the broader literature on conflicts between illness and identity [42-49]. This study suggests that avoidance coping may therefore be significant for individuals experiencing conflict between depressive symptoms and identity.

Also consistent with identity theories, participants reported that their use of avoidance strategies helped them to retain confidence in their ability to pursue their desired identity and goals [34-36]. However, as predicted by goal theory, which states that avoidance strategies work best with time-limited conflicts [37,38], avoidance often did not resolve their conflict and resulted in prolonged help-seeking delays. Participants reported increasing symptom severity and emotional distress as the delay period progressed. This is consistent with previous research that has identified help-seeking delays as a predictor of longer illness length and increased symptom severity at the start of treatment [14].

As avoidance became less effective at managing increasing symptoms and emotional distress, participants often discussed reaching a point of acceptance. At that point participants reduced their use of avoidance, and instead began to acknowledge the impact depression was having on their lives. Crucially, the acceptance process involved making concessions in identity and goals in order to incorporate depressive symptoms as part of identity (e.g., “I am someone who is depressed.”). This reduced the conflict caused by symptoms, and it was at this time that participants first considered seeking help. These findings raise the possibility that individuals with depression may need to make concessions in their identity and goals before seeking help. This process is consistent with studies which have demonstrated that people are more likely to enact behaviour that is congruent with their personal goals [50,51]. Further, in the identity literature the ability to incorporate illness into identity has been shown to predict help-seeking behaviour [42-44,46-49].

This may be an important consideration for interventions intended to encourage individuals to seek help. However, while goal theories predict that individuals will reorganise identity and goals and take action to resolve conflicts when they are long-lasting [37,38], very little is known about the way that individuals make the decision to do this. In this study, participants who had a history of previous help-seeking were able to resolve identity conflict more quickly than those without that history, and were able to seek help earlier. Several participants also reported that social support was instrumental in the resolution of identity conflict, and was predictive of earlier help-seeking. However, these were a small number of participants, reporting both supportive and unsupportive experiences with social support, and it is unclear from these results how social support was able to reduce identity conflict in these participants. It was interesting to note that social support did not emerge as having influenced help-seeking decisions in other participants, as previous research indicates that social support can facilitate help-seeking for mental health problems [52]. One way in which social support is thought to influence help-seeking is through changes in attitudes towards seeking help [52-54]. Future research exploring how individuals form and make changes in their attitudes to seeking help, and decide to make changes in their identity and goals, may reveal how help-seeking interventions can use social support and other mechanisms to increase help-seeking behaviour.

### Clinical implications

Help-seeking interventions that focus on reducing the costs and barriers of seeking treatment for depression may only be effective at increasing help-seeking behaviour in a sub-section of depressed individuals. Health care professionals and clinicians interacting with depressed individuals may benefit from talking to the individual about their concerns that receiving treatment may conflict with their goals and identity. Providing reassurance around these issues, and pointing out how treatment can help the individual retain and re-engage in important goals that support their identity may reduce emotional distress and improve willingness to engage in treatment. Likewise, future help-seeking interventions may benefit from incorporating mechanisms intended to reduce identity conflict, such as those that can support individuals to continue to pursue valued goals and maintain a positive identity alongside seeking treatment. Similar strategies are components of some psychological therapies, such as CBT, Behavioural Activation, and Acceptance and Commitment Therapy. Extending these into help-seeking interventions in public health campaigns may contribute to reductions in help-seeking delays, and improve the effectiveness of cost-benefit interventions.

### Limitations

This is an exploratory, qualitative study that provides an insight into the patient perspective on seeking help for depression. Future research is needed to investigate these findings further, including the use of quantitative methods, to identify specific mechanisms involved in the identity conflict process and assess the importance of identity in predicting help-seeking rates.

Future research should also seek to investigate these findings in a wider population of individuals. In particular, very few men volunteered to participate in this research, and their help-seeking narratives are therefore under-represented. As research frequently indicates that men are less likely to seek help for depression, greater understanding of the help-seeking process in men is greatly needed [55-58]. A recent review of this literature has argued that poor help-seeking rates may be in part due to men struggling to reconcile experiencing emotional difficulties with the expectations for the male gender role in industrialised societies [57]. Therefore the resolution of identity conflict following symptom onset may be a useful framework through which to understand poor help-seeking rates in men. Future research should therefore explore the extent to which men incorporate gender roles into their identity, and whether enhanced difficulties in resolving identity conflict contribute to poorer help-seeking behaviour in men. Due to greater interest from women in participating in this study, future research may therefore benefit from using purposive sampling to overcome difficulties in recruiting male participants.

Four participants in this study reported help-seeking delays of longer than ten years, which is not uncommon for help-seeking delays for MDD and other mental health disorders [8,11,12]. However, it should be noted that participants’ accounts, particularly when there is a long delay period, may contain some inaccuracies. Furthermore, only three participants in the sample had not sought help for depression at the time of interview, and so the findings may also have limited generalisability for individuals who do not seek help. These issues may represent inherent difficulties in recruiting participants to help-seeking research, and future researchers should therefore be mindful of these limitations when designing and interpreting the results of help-seeking studies.

Semi-structured interviews were employed to encourage participants to discuss their personal experiences in seeking help for MDD, as well as to facilitate discussion of factors suggested by the existing literature (e.g. difficulties recognising depressive symptoms). However, this approach, as well as the iterative adaptation of the schedule to focus on emerging themes, may have influenced the direction of the interviews and the analysis. Future studies may consider using an unstructured interview approach, which can also have the benefit of providing greater detail of participants’ experiences and their own terminology.

## Conclusions

This study presents a patient perspective of factors influencing the decision to seek help for depression. Participant accounts describe the way in which individuals recognise the onset of depressive symptoms, and provide support for a two-stage process model of help-seeking. These results enhance our understanding of the reasons that may underlie widespread help-seeking delays, and can be used to inform future research aimed at improving treatment access for depression.

## Abbreviations

MDD: Major Depressive Disorder.

## Competing interests

The authors declare they have no competing interests.

## Authors’ contributions

CF designed the study and conducted thematic analysis of the transcripts as part of her PhD research under the supervision of PF and HO. CF interviewed all participants. PF and HO performed second coding 10% of transcripts and contributed to the development of themes. All authors contributed to revising the manuscript. All authors read and approved the final manuscript.

## Pre-publication history

The pre-publication history for this paper can be accessed here:

http://www.biomedcentral.com/1471-244X/12/164/prepub
